# High concentrations of glucose reduce the oxidative metabolism of dog neutrophils *in vitro*

**DOI:** 10.1186/1746-6148-9-24

**Published:** 2013-02-06

**Authors:** Anelise M Bosco, Breno FM de Almeida, Priscila P Pereira, Luis G Narciso, Valéria MF Lima, Paulo C Ciarlini

**Affiliations:** 1 Department of Clinical, Surgery and Animal Reproduction, College of Veterinary Medicine of Araçatuba, São Paulo State University, Araçatuba,, SP, Brazil

**Keywords:** Superoxide, Respiratory burst, Programmed cell death, Leukocyte dysfunction, Hyperglycemia

## Abstract

**Background:**

Dogs are commonly affected by hyperglycemic conditions. Hyperglycemia compromises the immune response and favors bacterial infections; however, reports on the effects of glucose on neutrophil oxidative metabolism and apoptosis are conflicting in humans and rare in dogs. Considering the many complex factors that affect neutrophil oxidative metabolism *in vivo*, we investigated *in vitro* the specific effect of high concentrations of glucose on superoxide production and apoptosis rate in neutrophils from healthy dogs.

**Results:**

The capacity of the neutrophils to reduce tetrazolium nitroblue decreased significantly in the higher concentration of glucose (15.13 ± 9.73% (8 mmol/L) versus 8.93 ± 5.71% (16 mmol/L)). However, there were no changes in tetrazolium nitroblue reduction at different glucose concentrations when the neutrophils were first activated with phorbol myristate acetate. High concentrations of glucose did not affect the viability and apoptosis rate of canine neutrophils either with or without prior camptothecin stimulation. This study provides the first evidence that high concentrations of glucose inhibit the oxidative metabolism of canine neutrophils *in vitro* in a manner similar to that which occurs in humans, and that the decrease in superoxide production did not increase the apoptosis rate.

**Conclusions:**

A high concentration of glucose reduces the oxidative metabolism of canine neutrophils *in vitro*. It is likely that glucose at high concentrations rapidly affects membrane receptors responsible for the activation of NADPH oxidase in neutrophils; therefore, the nonspecific immune response can be compromised in dogs with acute and chronic hyperglycemic conditions.

## Background

Hyperglycemia occurs frequently in critically ill dogs hospitalized in intensive care units (16%), and their morbidity is directly associated with their blood glucose concentration [1]. In humans, functional defects in neutrophils that diminish the microbicidal and phagocytic function of the cells and increase the risk of bacterial infection are attributed to hyperglycemia [2]. The molecular, biochemical and cellular basis of this decrease in immunological defense is not well understood in humans [3], and the relationship between hyperglycemia and neutrophil dysfunction in dogs remains to be adequately investigated.

Reactive oxygen species (ROS), including superoxide anion and its derivatives, exert an important bactericidal function in neutrophils and are components of some of the conditions associated with the pathogenesis of diabetes [4]. However, the effect of hyperglycemia on neutrophil production of ROS is unclear. Some studies have reported increased ROS production in diabetic patients [5-7], and in healthy patients that underwent a hyperglycemic challenge [8]. The reduction of the neutrophil oxidative burst in patients with poorly controlled diabetes mellitus has also been described [9], but such alteration did not occur in patients with experimentally varied concentrations of blood glucose [10] and with acute, induced hyperglycemia [11].

There are many complex factors affecting the neutrophil respiratory burst *in vivo*, and different concentrations of blood glucose in patients can also affect the oxidative metabolism of neutrophils. Thus, to minimize these factors, *in vitro* protocols have been used to provide an adequate understanding of how glucose concentrations can affect the oxidative metabolism of neutrophils. Neutrophils from healthy individuals, when incubated with high concentrations of glucose *in vitro*, have lower superoxide anion production [12,2,13]. In humans, the *in vitro* inhibition of neutrophil oxidative metabolism seems to be dependent on glucose concentration. Perner *et al.*[14] incubated neutrophils from healthy patients in solutions containing 5 mmol/L, 10 mmol/L and 25 mmol/L of glucose and verified 50% inhibition of superoxide production in the highest glucose concentration.

Nielson *et al.*[12] suggest that the suppressing effect of glucose on the oxidative metabolism of neutrophils is due to protein glycosylation. Monosaccharides such as glucose, mannose and fructose form Schiff-base adducts with proteins, which cause inhibition of neutrophil function. Four mechanisms underlie the oxidative stress associated with hyperglycemia: increased polyol pathway flux, increased advanced glycation end product (AGE) formation, activation of protein kinase C (PKC) isoforms, and increased hexosamine pathway flux [15].

There is evidence that oxidative stress in diabetics increases apoptosis of neurons [16], endothelial cells [17], pancreatic cells [18], mesangial cells [19], and renal podocytes [20]. However, the relationship between hyperglycemia and neutrophil viability needs further study. According to Tennenberg *et al.*[21], high concentrations of blood glucose decrease the functional longevity of human neutrophils and increase neutrophil clearance from infected sites, possibly contributing to the increased susceptibility to and severity of infections in diabetic patients. In contrast, Turina *et al.*[22] observed no alteration in human neutrophil apoptosis when exposed to high concentrations of plasma glucose. In diabetic rats, the glycolytic metabolism and the protector effect of glutamine on neutrophils is altered, accelerating apoptosis and increasing susceptibility to infections [23].

Although dogs are commonly affected by diabetic hyperglycemia and are considered the best animal model for the study of human diabetes [23], there are few studies examining the effect of glucose on neutrophil oxidative metabolism and apoptosis in dogs. The decrease in NADPH oxidase activity seen in the neutrophils of dogs fed high levels of galactose results from excessive consumption of enzyme due to increased polyol pathway activity [24]. In this circumstance, excessive consumption of NADPH decreases superoxide production and compromises the bactericidal function of neutrophils. A diabetic retinopathy, similar to that which occurs in humans, was induced in dogs fed a 30% galactose diet. When isolated neutrophils from healthy dogs were incubated with a similar concentration of galactose there was no alteration in their apoptosis rate; however, *in vivo* evaluation of neutrophils revealed a lower apoptosis rate and higher adhesion in galactose-fed dogs [25].

*In vitro* studies are necessary to evaluate the specific effects of glucose on neutrophil oxidative metabolism and apoptosis, especially in diabetic dogs and hyperglycemic conditions. The aim of this study was to test, *in vitro*, whether high concentrations of glucose inhibit the oxidative metabolism of isolated neutrophils from healthy dogs, and also to evaluate the viability and apoptosis rate of these cells when incubated in different concentrations of glucose.

## Results

### A high glucose concentration inhibits the oxidative metabolism of canine neutrophils

The interference of glucose with the oxidative metabolism of human neutrophils has been described [2,12,14]. This interference was evaluated in dogs using the NBT reduction test to quantify the superoxide production of neutrophils from healthy dogs in RPMI medium containing a high concentration of glucose.

Neutrophils incubated in pure RPMI medium reduced significantly more NBT than neutrophils incubated in RPMI with a high concentration of glucose (15.13 ± 9.73% (8 mmol/L) versus 8.93 ± 5.71% (16 mmol/L)). Thus, similar to the effects seen in human neutrophils *in vitro*[14], superoxide production decreased in canine neutrophils in the presence of a high concentration of glucose. However, no inhibitory effects on neutrophil oxidative metabolism were observed when the cells had been previously activated with PMA (Table 1).

**Table 1:** Oxidative metabolism of neutrophils from healthy dogs at two different concentrations of glucose

**Glucose concentration**	**Percentage of neutrophils reducing NBT**	
	**Spontaneous**	**PMA activation**
**8 mmol/L**	15.13 ± 9.73^a^	73.66 ± 8.52^a^
**16 mmol/L**	8.93 ± 5.71^b^	72.20 ± 9.71^a^
**p-value**	0.0304	0.6444

* Different letters in the same column indicate a statistically significant difference.

### Maintenance of neutrophil viability during incubation with a high concentration of glucose

We compared the viability and apoptosis rate of neutrophils isolated from healthy dogs and incubated in pure RPMI media or in RPMI media with addition of glucose. The protocol for neutrophil isolation and incubation in RPMI media did not compromise cell viability, even though the glucose concentration of RPMI media is 8 mmol/L, which is higher than that of normal dog plasma (4–6 mmol/L) (Table 2). When apoptosis was stimulated with CAM, there was a decrease in viability associated with increased neutrophil apoptosis in both the pure and the glucose-enriched media. RPMI enriched with glucose resulted in a numerical increase in the apoptosis rate; however, this difference was not statistically significant.

**Table 2:** Viability and apoptosis of neutrophils from healthy dogs incubated at two glucose concentrations

**Glucose concentration**	**Percentage of neutrophils**							
	**Spontaneous**				**CAM activation**			
	**Viability**	**Initial apoptosis**	**Final apoptosis**	**Total apoptosis**	**Viability**	**Initial apoptosis**	**Final apoptosis**	**Total apoptosis**
**8 mmol/L**	97.93 ± 2.44	0.19 ± 0.19	0.19 ± 0.12	0.38 ± 0.23	69.91 ± 15.34	5.87 ± 4.77	19.16 ± 13.09	25.04 ± 14.60
**16 mmol/L**	97.76 ± 1.90	0.22 ± 0.23	0.26 ± 0.24	0.49 ± 0.43	67.73 ± 12.31	6.54 ± 5.72	22.21 ± 15.04	28.76 ± 12.66
**p-value**	0.8221	0.6812	0.4202	0.3593	0.6508	0.7132	0.5327	0.4332

## Discussion

Multiple cellular factors have been associated with neutrophil dysfunction in hyperglycemic conditions. These include nicotinamide [9], protein components of NADPH oxidase [26], intracytoplasmic cytokines [27], chemokines [28], expression of pro-inflammatory genes [11], and alterations in several different mechanisms of cellular activation as well as modifications of proteins and lipids [29]. However, the complete mechanism underlying glucose-associated neutrophil dysfunction is still unknown.

In this study, we verified *in vitro* that neutrophils of healthy dogs decrease superoxide production when incubated in a high concentration of glucose (16 mmol/L). Similar results were observed in human neutrophils incubated with elevated concentrations of glucose such as ≥11 mmol/L [12], ≥ 13.8 mmol/L [2] and 25 mmol/L [14]. These results reinforce the affirmation of Ionut *et al.* (2010) [30] that the dog is the most suitable model for the study of human diabetes.

The *in vitro* inhibitory effect of glucose on the oxidative metabolism of canine neutrophils has also been observed in humans *in vivo*[9]; however, some studies did not find alterations in the oxidative metabolism of diabetic patients [10,11], while others found increased oxidative metabolism [8,5-7]. This discrepancy among the results of studies that evaluated neutrophil oxidative metabolism may be due to the heterogeneity of their protocols; different concentrations of glucose were used, various stimuli were used to activate the cells, and oxidative metabolism was assessed by different methods [3]. Although the NBT method is not able to determine the precise concentration of superoxide produced, in this study and in others [31-34] it was possible to assess neutrophil oxidative metabolism using this method.

Neutrophil activation occurs by a variety of distinct mechanisms of signal transduction. Some neutrophil activators depend on complement (calcium ionophore A23187), while others require opsonization (zymosan) and others, such as N-formyl-methionine-leucine-phenylalanine (fMLP), depend on specific protein receptors [35]. In this study, the potent neutrophil activator PMA was used. PMA does not require receptors to activate NADPH oxidase, instead inducing superoxide production directly by protein kinase C activation [36]. According to Mcmanus *et al.* (2001) [3], in diabetic patients neutrophil activation occurs only in the presence of stimuli that initiate signal transduction via G-protein coupled receptors, thus it does not occur in the presence of PMA. This may explain why excessive glucose in the media did not alter the oxidative metabolism of neutrophils activated with PMA, and suggests that the inhibition of oxidative metabolism observed in the trials without PMA is due to a failure in G-protein coupled signal transduction, which is responsible for NADPH oxidase activation.

There is evidence that a high concentration of glucose decreases neutrophil functional longevity in humans and increases neutrophil clearance from infected sites, possibly contributing to the increased susceptibility to and severity of infections in diabetic patients [21]. The mechanisms related to the acceleration of apoptosis during hyperglycemia are associated with decreased resistance to oxidative stress, increases in protein glycosylation, and decreased protective effect of glutamine [23].

Oxidative stress associated with short-term hyperglycemia increases the apoptosis rate of neurons [16] and renal podocytes [20]. Conversely in our study, neutrophils maintained for a short period (4 h) in glucose-rich media did not have an increased apoptosis rate. Similar results were observed in human neutrophils incubated for 24 h with high (100 mg/dL) and low (10 mg/dL) concentrations of glucose [22]. Similarly, no acceleration of neutrophil apoptosis was observed in diabetes-induced dogs [25] and rats [37]. Therefore, it is reasonable to assume that the varying results regarding the assessment of neutrophil apoptosis in short-period experiments are due to a pro-apoptotic effect of glucose that only occurs in conditions of persistent hyperglycemia.

## Conclusions

This study provides the first evidence that high concentrations of glucose inhibit the oxidative metabolism of canine neutrophils *in vitro*, and that this inhibition does not alter the apoptosis rate and viability of the neutrophils. High concentrations of glucose may rapidly affect the membrane receptors responsible for the activation of NADPH oxidase; in this way even short-term hyperglycemia can compromise the oxidative metabolism of canine neutrophils. However, further investigations are required to evaluate the clinical implications of these results.

## Methods

### Animal selection

Seventeen adult mongrel dogs of both sexes were used. Only dogs with no abnormalities on physical examination and laboratory tests (hemogram, plasma fibrinogen, urinalysis, plasma albumin, urea, creatinine, uric acid, cholesterol, glucose, and fructosamine) were included. The region is endemic for leishmaniasis, and dogs were selected for the study only after negative serological testing. No animal that had recently received treatment with any drug was included in the experiment. The study was approved by the Animal Experimentation Ethics Committee of the Faculty of Veterinary Medicine of São Paulo State University and samples were obtained with the consent of the owners.

### Samples collection and maintenance

Whole blood samples were collected using disposable needles and syringes and kept in heparinized sterile glass tubes with 10 U of heparin (Vacutainer plus plastic Heparin, Cod. 367993, Becton-Dickson, New Jersey, USA) per 1 mL of whole blood. All the samples were protected from light and kept at 4°C for a maximum period of 2 h before analysis. Urine samples were collected by cystocentesis and were also kept at 4°C and protected from light until analysis.

### Biochemical analyses, hemogram and urinalysis

All biochemical tests were performed using commercial reagents (BioSystems Reagents, Barcelona, Spain) at 37°C in an automated analyzer (Automatic Analyzer BTS, Mod. 370 plus, BioSystems) that had been previously calibrated with a commercial calibrator, and reaction quality was monitored with control levels I and II (Biosystems, Barcelona, Spain). Plasma urea concentration was determined using the urease/glutamate dehydrogenase-coupled UV enzymatic assay, creatinine using the alkaline picrate kinetic assay, albumin using the bromocresol green assay, uric acid using the uricase/peroxidase enzymatic assay, cholesterol and glucose by the enzymatic oxidase/peroxidase method, and fructosamine by the colorimetric method of NBT reduction.

White blood cell counts were performed in an automated veterinary blood cell counter (Veterinary hematological electronic cell counter, Mod. CC-530, CELM, São Paulo, Brazil). Differential white cell counts were accomplished using optical microscopy by the differentiation of 100 white cells in a blood smear stained with panoptic dye (InstantProv, Newprov, Pinhais-PR). Plasma fibrinogen was estimated indirectly by refractometry (Refratometre ATAGO® SPR-T2, ATAGO Co., LTD, Japan) using the heat precipitation method (56 – 58°C).

Urinalysis was performed as described by Osborne *et al.* (1995) [38] using commercial reagent strips for chemical analysis (Combur 10 test®, Roche, Mannheim, Germany) and refractometry for density determination.

### Neutrophil isolation

A double gradient separation technique was used to isolate neutrophils. Four mL of heparinized whole blood (10 IU/mL) were transferred to sterile polypropylene conical tubes containing equal volumes (3 mL) of Histopaque-1119 and 1077 (Sigma, St. Louis, MO, USA). Following centrifugation at 340 G for 30 min, the layer of polymorphonuclear (PMN) cells was aspirated and washed twice with aqueous ammonium chloride (0.14 M) to ensure complete lysis of the residual erythrocytes. Next, the sample was centrifuged (100 G) for 5 min in Hanks' balanced salt solution (HBSS) (Sigma) without Ca2+ and Mg2+ and 1 mL of RPMI media (Sigma) was added to the cell sediment. Cell concentration was determined in a hemocytometer and cell viability was estimated by the blue trypan dye exclusion method. The sample of isolated PMNs was diluted in RPMI to obtain a final cell concentration of 10^6^ mL, with the purity and viability of neutrophils equal to or more than 90 and 95%, respectively.

### Determination of neutrophil superoxide production

Neutrophil superoxide production was estimated by the nitroblue tetrazolium (NBT) reduction test. Briefly, 100 μL of the neutrophil suspension (10^6^/mL) was incubated in two sterile microtubes with 50 μL of 0.2% NBT solution in saline. For the stimulated test, 1 μL (535 μmol/L) of PMA (Phorbol 12-myristate 13-acetate, Cat. P8139, Sigma) was added. After incubation in a thermal shaker at 37°C for 10 min, the samples were cytocentrifuged at 400 G for 5 min and then stained with panoptic dye. The percentage of positive cells was assessed by counting 100 cells.

### Determining the viability and apoptotic index of the neutrophils

The percentage of apoptotic cells and viable neutrophils was determined using the Annexin V-PE assay Kit (Guava Nexin Kit, Guava Technologies, USA). The procedure for each test and proper instrument performance were verified in accordance with the manufacturer’s recommendations. Briefly, two samples of 100 μL of neutrophils (10^6^/mL) suspended in RPMI were incubated at 37°C for 1 h with 100 μL of RPMI medium (CAM absent) and with 100 μL of camptothecin (19.8 mmol/L, Sigma). Following incubation, 100 μL of each sample was transferred to a microtube with 100 μL of annexin V-PE reagent and incubated in the dark at room temperature for 10 min. In a capillary flow cytometer EasyCyte mini® (Guava, Hayward, CA) 10,000 events were acquired, and viable, apoptotic populations of neutrophils were delineated using a specific computer program (Software Express Plus® Guava, Hayward, CA).

### *In vitro* experimental design to test the hypothesis that glucose alters the oxidative metabolism and apoptosis of neutrophils from healthy dogs

Neutrophils were isolated from 17 healthy dogs and incubated in RPMI with two different concentrations of glucose (8 and 16 mmol/L). After 2 h of incubation, the superoxide production and the apoptosis rate of the neutrophils were evaluated in spontaneous and stimulated trials as described above.

### Statistical analysis

Statistical tests were performed after testing the distributions of the variables for normality (Kolmogorov-Smirnov test). Differences between control and hyperglycemic groups were assessed using paired t-tests, with *P* values of ≤0.05 considered significant. SAS software (SAS Institute Inc., The SAS System, release 9.2., Cary: NC, 2008) was used for all statistical analyses.

## Abbreviations

AMB: Anelise Maria Bosco; BFMA: Breno Fernando Martins de Almeida; PPP: Priscila Preve Pereira; LGN: Luis Gustavo Narciso; VMFL: Váleria Marçal Felix de Lima; PCC: Paulo César Ciarlini.

## Competing interests

The authors declare that they have no competing interests.

## Authors’ contributions

AMB: Principal investigator, performed the experiment, analyzed the data, mainly wrote the paper; BFMA: analysis and interpretation of the data; PPP: analysis and interpretation of the data; LGN: conduction of the study, interpretation of the data; VMFL: substantial contribution to acquisition of the data for the flow citometry analysis; PCC: statistical analyses, interpretation of the data, supervised the study. All authors participated in the writing of the paper and have read and approved the final manuscript.
